# Analysis of the Registration Information on Interventions of Acupuncture and Moxibustion Trials in the International Clinical Trials Registry Platform

**DOI:** 10.1155/2018/1054629

**Published:** 2018-01-15

**Authors:** Yali Liu, Wenjie Chen, Yingxin Tan, Xingyue Yang, Jia Liu, Tingting Lu, Shiyan Yan, Liyun He, Baoyan Liu

**Affiliations:** ^1^Institute of Basic Research in Clinical Medicine, China Academy of Chinese Medical Sciences, Beijing 100700, China; ^2^Postdoctoral Research Station, China Academy of Chinese Medical Sciences, Beijing 100700, China; ^3^Evidence Based Medicine Center, School of Basic Medical Sciences, Lanzhou University, Lanzhou, Gansu 730000, China; ^4^The First Clinical Medical College of Lanzhou University, Lanzhou, Gansu 730000, China; ^5^Beijing University of Chinese Medicine, Beijing 100700, China; ^6^China Academy of Chinese Medical Sciences, Beijing 100700, China

## Abstract

**Purpose:**

To analyze and compare the clinical registration information about acupuncture and moxibustion for intervention characteristics.

**Methods:**

Clinical trials from the International Clinical Trials Registry Platform of the World Health Organization in acupuncture and moxibustion were comprehensively collected from 2013 to 2015; data were independently screened and extracted by two retrievers, and relevant data involving either basic descriptions or intervention characteristics were analyzed.

**Results:**

425 acupuncture and moxibustion registered clinical trials were included; 88.00% (374/425) were designed as controlled studies, among which 38.59% (164/425) had sham acupuncture as the control group. The most common diseases were pain-related at approximately 19.29% (82/425) of trials. Reports on the intervention information in these acupuncture and moxibustion clinical studies were not sufficiently presented; these reports included the reporting of names of points (39.8%), the method of needle stimulation (32.5%), needle type (29.6%), needle retention time (34.1%), the number of treatment sessions (22.4%), and the frequency and duration of treatment sessions (38.1%).

**Conclusion:**

The registration information for the clinical trials of acupuncture and moxibustion was quite low according to this investigational study. Steps should be taken to improve the quality of acupuncture and moxibustion registration information.

## 1. Introduction

Acupuncture and moxibustion have been practiced in China for more than 3000 years and are considered to be complementary and alternative therapy [1]. Benefits from these therapies include relative safety, effectiveness, feasibility, and economic wellbeing. Since 1979, the World Health Organization (WHO) has promulgated 43 diseases with indications applicable to acupuncture and moxibustion [2]. Therefore, these traditional Chinese therapies have become publicly acceptable in clinical practice in the West [3]. 

Since the 20th century, acupuncture and moxibustion clinical trials have progressed into a bustling and booming era not only in the quantity of studies but also in quality; in addition, the diseases included in clinical trials have expanded over these decades [4, 5]. Many problems and concerns manifested during this time period; for example, there was a shortage of well-designed and strictly implemented clinical studies [6]. Evidence from these clinical studies could not be cited and generalized to improve acupuncture and moxibustion practice. The barrier has been diminishing with solid evidence.

In November 2004 in Mexico City, the WHO was regarded as the lead organization in establishing the International Clinical Trials Registry Platform (ICTRP) [7]. This milestone event was a consensus decision during the Ministerial Summit on Health Research [8]. It was in May 2007 when the ICTRP officially broke ground [9], and the importance of trial registration has been gradually accepted [10–12]. To date, 16 data providers, including the United States and China, have been authorized as the first registration institutions of the WHO ICTRP [13]. In May 2007, the WHO Trial Registration Data Set (TRDS) was announced, setting a minimum of 20 items for trial registration [14]. International clinical trial registration has been implemented to a large extent to improve the quality of clinical trials, as well as form a transparent presentation of trial progress and execution [15].

This study aims to collect and analyze the 20 items of the WHO TRDS and other registry information from the 16 clinical trial register centers of the WHO ICTRP; provide an overview of the registered acupuncture-moxibustion clinical trials for manipulation specifications and reporting performance; and propose the necessity to establish the Acupuncture-Moxibustion Clinical Trial Registry (AMCTR) center.

## 2. Materials and Methods

### 2.1. Inclusion/Exclusion Criteria

All acupuncture and moxibustion clinical trials registered in the WHO ICTRP were eligible for inclusion. No criteria for disease were applied, nor were criteria applied for participant age, sex, and ethnicity; the studied interventions were acupuncture and moxibustion, including* Filiform needle, percussopunctator, Auricular Acupuncture, or transcutaneous electrical acupoint stimulation*, indicated as the major medical treatment. The control group for any comparison was placebo, no intervention, or another intervention. There was no definition for endpoint outcomes. Exclusion criteria included incomplete registry information, no information indicating that acupuncture or moxibustion had been used to intervene in the disease studied or that acupuncture or moxibustion were only used as a secondary concomitant therapy.* Acupuncture, needling, acupressure, moxibustion, auriculotherapy, *and* acupoint* were used as keyword search terms, regardless of the initial registry language.

### 2.2. Database Sources

Data from the WHO ICTRP (http://apps.who.int/trialsearch/Default.aspx) registry records between 2013 and 2015 were used. This registry platform included Australia, New Zealand, China, Korea, United States, India, Cuba, Germany, Iran, United Kingdom (UK), Japan, Pan African Region, Sri Lanka, Netherlands, Belgium, and the European Union (EU) Region. The specific names of the registries are as follows: Australian New Zealand Clinical Trials Registry (ANZCTR, Australia, and New Zealand), Chinese Clinical Trial Register (ChiCTR, China), Clinical Research Information Service (CRIS, Republic of Korea), Clinical Trials.gov (United States), Clinical Trials Registry, India (CTRI, India), Cuban Public Registry of Clinical Trials (RPCEC, Cuba), German Clinical Trials Register (DRKS, German), Iranian Registry of Clinical Trials (IRCT, Iran), ISRCTN.org (British), Japan Primary Registries Network (JPRN, Japan), Pan African Clinical Trial Registry (PAC-TR, Africa), Sri Lanka Clinical Trials Registry (SLCTR, Sri Lanka), the Netherlands National Trial Register (NTR, Netherlands), Brazilian Clinical Trials Registry (ReBec, Brazil), EU Clinical Trials Register (EU-CTR, EU), and Thai Clinical Trials Registry (TCTR, Thailand).

### 2.3. Data Extraction

Using a predefined data extraction form that collected information for this study, two evaluators independently extracted data (Wenjie Chen and Yingxin Tan); disagreements in the data extraction were resolved by another evaluator (Yali Liu) after further consultation. The content of the data extraction forms was categorized into the following sections.

#### 2.3.1. Basic Registry Information on the 16 Clinical Trial Registry Centers

This information included the name of the registry platform, nationality/region, website, language, the date in which the platform was established, the setup and disposition of the 16 first-rate study centers worldwide, and the 20 items in the World Health Organization (WHO) Trial Registration Data Set (TRDS).

The categories for the registry information were formulated by the 16 ICTRP clinical study centers; these included the registered title for the platform, update time, registry time, status of the registry number, information on the applicant, approval-related report from the institutional ethics board, information on the study-center that conducted the designated trials, funding and financial resources, study design (disease, study category/type, purpose, design, methodology, inclusion and exclusion criteria, interventions, outcomes, and endpoints), study time (initiation, duration, and close-out), designated study sites, human histology samples collected, subject recruitment and enrollment, study data management institution, and any commissioned/contracted data analysis companies/agents.

#### 2.3.2. Basic Information Registered Clinical Trials in the Acupuncture and Moxibustion Fields

These fields involved registry time, the name of the clinical trials registry platform, study type, sample size, the object disease/symptoms, intervention(s), and endpoint outcomes.

#### 2.3.3. Specific Information on Interventions in Registered Clinical Trials in the Acupuncture and Moxibustion Fields

Based on the Standards for Reporting Interventions in Clinical Trials of Acupuncture (STRICTA) [16], the data extraction form was used to investigate the manipulation procedures. The information collected included the number of needle insertions per subject per session, names (or location if no standard name) of points used, depth of insertion, response sought, needle stimulation, needle retention time, needle type, and the registry information on sham acupuncture.

### 2.4. Data Analysis

Microsoft Excel (Version 2007) was used to analyze the coded data items for any statistically significant differences. Categorical data is presented as number (*n*) and percent (%).

## 3. Results

### 3.1. Basic Information of the Registered Clinical Trial Centers of ICTRP

To date, 16 registries have been designated by the WHO ICTRP as first-rate clinical trial registry centers which cover all six continents. These are summarized in Table 1.

### 3.2. Categorization of the Registry Information Designated by the ICTRP Clinical Trial Registry Centers

There are 20 items in the TRDS stipulated by the WHO ICTRP that are reported by the first sixteen registry platforms, as shown in Table 2. Moreover, specific registry information from these centers supplements the fundamental TRDS. In Table 3, some supplemental data are included, except for the 20 items in the WHO TRDS; in this study, a total of 45 registry items are included. It is notable to mention that the ANZCTR had defined more than 30 items for its registry information; other centers have sets of items that vary from 15 to 25.

### 3.3. Basic Registry Information for Acupuncture and Moxibustion Clinical Trials

We reviewed 425 acupuncture and moxibustion clinical trials that had completed registration between 2013 and 2015. Among them, 124 (29.18%) were registered in 2013, 123 (28.94%) were registered in 2014, and 178 (41.89%) were registered in 2015. The studies that were registered in the WHO ICTRP were mainly interventional studies (89.18%, 379/425); only 3.53% (14/425) were observational studies; 88.00% (374/425) were controlled studies and 164 (38.59%) were compared with sham acupuncture; 187 and 50 studies recruited participants from China (including Hong Kong and Taiwan) and the United States, respectively. The largest sample size was 1000 subjects (Table 4). Clinical Trials.gov, ChiCTR, and CRIS had the most clinical studies (Table 5). Pain symptoms were the main indication for treatment with acupuncture or moxibustion, comprising 82 (19.29%) of the studies; the other diseases and symptoms were neoplasm (7.29%, 31/425), stroke (4.00%, 17/425), arthritis (3.53%, 15/425), depression (3.06%, 13/425), and insomnia (2.59%, 11/428) (Table 6).

### 3.4. Characteristics of the Acupuncture and Moxibustion Trials in the WHO ICTRP

As summarized in Table 7, there were no sufficiently specific descriptions of acupuncture and moxibustion manipulation or the procedures in these reports of the clinical trials registered between 2013 and 2015. These trials mainly covered Acu-point [39.8% (169/425)], the method for acupuncture stimulation [32.4% (138/425)], the instrumental needle type [29.6% (126/425)], needling keep-in time [34.1% (145/425)], unit number for acupuncture therapy [22.4% (95/425)], therapeutic frequency, and the timing of each acupuncture scheme/regimen [38.1% (162/425)]. Among the countries of recruitment in these platforms, China represented 1.6% (3/187), 34.8% (65/187), 10.7% (20/187), 8.0% (15/187), 39.6% (74/187), 29.4% (55/187), 17.1% (32/187), 16.0% (30/187), and 32.1% (60/187), respectively. Fewer trials reported other details of the acupuncture intervention. For example, only 11.8% (50/425) of the trials reported anchoring depth, 8.9% (38/425) reported if a somatic response was triggered (such as specific self-sensual response from a receiver), and 4.9% (21/425) reported the number of needles. The manipulation in these registered studies was mainly manual acupuncture (55.06%, 234/425); in the others it was electrical stimulation (22.59%, 96/425), acupressure (4.23%, 18/425), auricular acupuncture (3.53%, 15/425), dry needling (4.70%, 20/425), laser acupuncture (4.23%, 18/425), moxibustion (2.35%, 10/425), auricular acupressure (0.47%, 2/425), thermotherapy (0.70%, 3/425), and pharmaco-acupuncture (0.23%, 1/425) (Table 8).

Among the 425 trials, 164 (38.59%) trials used sham acupuncture as a control, but specific information, such as the application materials (0.61%, 1/164), needle stimulation (14.02%, 23/164), depth of insertion (11.59%, 19/164), and needle retention time (0.61%, 1/164), was reported at a low rate; 61 (32.70%) trials did not report any details about the sham acupuncture (Table 9).

## 4. Discussion

### 4.1. Current Status of the Registration Information in the WHO ICTRP

In this overview, we have compared the registry items of the 16 clinical centers that were designated according to the recommendations of the WHO ICTRT. Generally, the minimum 20 items in the WHO TRDS were followed; however, information outside of these 20 items varied widely between different countries. The main information reported was a brief summary, purpose, trial completion time (registry update date, time to trial termination), methodologic details (inclusion criteria, age of the subjects, study phase, and design including groups/distribution/blinding of subjects and blinding of groups/interventions/drug names), and other information (dropouts, ethical issues, data monitoring committee, and updates on important information).

### 4.2. Deficiencies in the Characteristics of Clinical Trials of Acupuncture and Moxibustion Registered in the ICTRP

Acupuncture and moxibustion are a core part of traditional Chinese medicine and are also very important in complementary and alternative medicine. Compared with surgery and other modern medical interventions, acupuncture and moxibustion are characterized by obvious differences. To standardize clinical study reporting on acupuncture and moxibustion, STRICTA and STRICTOM (Standards for Reporting Interventions in Clinical Trials of Moxibustion) have been issued as an extended section in the CONSORT statement [17–19].

Six criteria have been set as STRICTA items: acupuncture rationale, details of needling, treatment regimen, other components of treatment, practitioner background, and control or comparator interventions [17]. In this study, a data extraction form was designed based on the STRICTA to collect and analyze the registration information. As the results indicated, necessary information, such as instrumental needle type, name of points used, depth of insertion, method of needle stimulation, needle retention time, response sought, and treatment sessions, was incompletely reported (less than 40% according to our analysis). However, this information needs to be described in detail because it is an important component of integrative therapy, directly influencing the authenticity and reliability of studies.

Controlled studies are fundamental to assessing effects in clinical trials. The purpose of controlled studies is to minimize confounding factors and provide a reliable, cogent conclusion. Because of their unique manipulation process, acupuncture and moxibustion have encountered difficulties in developing and validating placebo needles as appropriate controls [20]. Researchers designate sham acupuncture as a control group, such as sham treatment with invasive skin or needling at inappropriate points or at nonacupuncture points, needling with blunt needles, sham laser, and sham electroacupuncture [16, 21–24]. However, the rationale for these simulation designs and their anticipated placebo-effect is closely related to conscientious pseudo-manipulation and, consequently, rigid implementation. Detailed reports are needed about sham acupuncture materials, sham-acupoint, or locations without standard names, manipulation methods, and procedures (stimulation methods, depth of insertion). Moreover, the rationale for sham-needling or its purported placebo-effect should also be explained; otherwise, the reader of the study cannot comprehend or be persuaded to trust the sham-needling maneuvers to enhance the accuracy of measuring intervention effectiveness. Among the 164 trials reporting the use of sham acupuncture, some necessary information such as materials, stimulation methods, depth of insertion, and duration are not available because of the low incidence of reporting.

The reporting rate of the characteristics of acupuncture and moxibustion in the WHO ICTRP for both Chinese and clinical trials and those from other countries was low. The reasons may be as follows: (1) all 16 clinical trials registry centers were founded based on the mode or convention of Western-medicine-oriented registry format design, which may not have sufficiently considered the characteristics/specifications of acupuncture and moxibustion; (2) clinical trials reported and delineated acupuncture interventions in a meticulous manner, and completing registration information was done with little detail; (3) the inclusion and exclusion criteria for the clinical trials, such as the PICO, have not been included in the registration forms even though they might be well described in the trial protocols because of the limitation of the module in clinical register platform.

To summarize, some necessary information may have been missed in the registered clinical trials. This implies that steps of the registration process may not have been highly valued and, consequently, led to deficiencies in these reports.

### 4.3. Considerable Work Needs to Be Done to Improve Methodological and Reporting Quality of Acupuncture and Moxibustion Clinical Trials

As developments in clinical epidemiology and evidence-based medicine continue, some methods of interventional investigation to guide clinical practice have been modified/updated into more incisive study designs. Clinical studies in acupuncture and moxibustion have been affected by the introduction of some clinical study methods, as demonstrated by the increased number of trials and the reporting quality; although there was steady progress, some remarkable redirected attention could be identified.

However, problems in practice and in studies inhibit substantial improvement and adaptation to modern medicine. Overall, rigidly controlled studies resulting in a highly credible publication comprised only a small number of the total [6]. Second, many trials have deficiencies and deviate from the clinical study methodology, even these that were labelled as systematic assessments and randomized controlled trials. Specific examples include lack of statistical calculation of sample size, incomplete analysis of baseline data, fragmented reporting on follow-up, or insufficiently scheduled visits, which can seriously undermine the reliability of clinical evidentiary studies [25]. Third, there are still large gaps between the clinical evidence and the clinical practice of acupuncture and moxibustion.

Therefore, a challenging and long-term goal to be undertaken is promoting the standardization of acupuncture and moxibustion clinical trial registries. The current principles and guidelines of institutions for clinical registration trials are skewed in favor of western medical drugs or surgical interventions, ignoring the special characteristics of acupuncture and moxibustion, which are founded on the Meridian-Collateral Theory.

The Acupuncture-Moxibustion Clinical Trials Registry (AMCTR), making the second-tier of the WHO ICTRP, has been established to regulate clinical trials of acupuncture and moxibustion [26]. This registry center will be obliged to improve the global mechanism, promote data collection, facilitate data sharing, regulate specific trial studies, and assure publicized honesty and integrity of acupuncture and moxibustion studies. It will also provide a registry service in Chinese regions, as well as for worldwide interest.

### 4.4. Limitations

Limitations include the following: (1) the study was only based on data collection from 2013 to 2015 from the WHO ICTRP register trials in acupuncture and moxibustion; (2) the main focus is on registering information from designated sources instead of acquiring specific study protocols; and (3) incomplete information from these registries could influence the results.

## Figures and Tables

**Table 1:** The characteristic information of platforms in ICTRP.

Platform	Countries/District	Internet site	Language	The time of establishment
ANZCTR	Australia/New Zealand	http://www.anzctr.org.au/	English	2003
ChiCTR	China	http://www.chictr.org.cn/	English, Chinese	2005
CRIS	Kore	http://cris.nih.go.kr/	English, Korean	
Clinical Trials.gov	United States	http://www.clinicaltrials.gov/	English	1988 1997
CTRI	India	http://www.ctri.nic.in/	English	2005
RPCEC	Cuba	http://registroclinico.sld.cu/	English, Spanish	
DRKS	Germany	http://www.germanctr.de/	English, German	2008
IRCT	Iran	http://www.irct.ir/	English, Arabic	
ISRCTN.org	United Kingdom	http://www.isrctn.org/	English	2007
JPRN	Japan	http://rctportal.niph.go.jp/	English, Japanese	1988
PAC-TR	Africa	http://www.pactr.org/	English	2005
SLCTR	Sri Lanka	http://www.slctr.lk/	English	
NTR	Netherlands	http://www.trialregister.nl/	English	
ReBec	Brazil	http://www.ensaiosclinicos.gov.br/	English, Portuguese	
EU-CTR	Europe	https://www.clinicaltrialsregister.eu/	English	2004
TCTR	Thailand	http://www.clinicaltrials.in.th/	English, Thai	

**Table 2:** The WHO Trial Registration Data Set (TRDS).

20 items	ANZCTR	ChiCTR	CRIS	Clinical Trials.gov	CTRI	RPCEC	DRKS	IRCT	ISRCTN.org	JPRN	PAC-TR	SLCTR	NTR	ReBec	EU-CTR	TCTR
Primary register and trial IDb	Y	Y	Y	Y	Y	Y	Y	Y	Y	Y	Y	*Y*	Y	Y	Y	Y
Date of registration in primary registry	Y	Y	Y	Y	Y	Y	Y	Y	Y	Y	Y	*Y*	Y	Y	Y	Y
Secondary IDb	Y	Y	Y	Y	Y	Y	Y	N	Y	Y	Y	*Y*	Y	Y	Y	Y
Source(s) of monetary or material support	Y	Y	Y	Y	Y	Y	Y	Y	Y	Y	Y	*Y*	Y	Y	Y	Y
Primary sponsor	Y	Y	Y	Y	Y	Y	Y	Y	Y	Y	Y	*Y*	Y	Y	Y	Y
Secondary sponsor(s)	Y	Y	Y	Y	Y	Y	Y	Y	Y	Y	Y	*Y*	N	Y	Y	Y
Contact for public queries	Y	Y	Y	Y	Y	Y	Y	Y	Y	Y	Y	*Y*	Y	Y	Y	Y
Contact for scientific queries	Y	Y	Y	Y	Y	Y	Y	Y	Y	Y	Y	*Y*	Y	Y	Y	Y
Public title	Y	Y	Y	Y	Y	Y	N	Y	N	Y	Y	*Y*	Y	Y	Y	Y
Scientific title	Y	Y	Y	Y	Y	Y	Y	Y	Y	Y	Y	*Y*	Y	Y	Y	Y
Countries of recruitment	Y	Y	Y	Y	Y	Y	Y	Y	Y	Y	Y	*Y*	N	Y	Y	Y
Health condition(s) or problem(s) studied	Y	Y	Y	Y	Y	Y	Y	Y	Y	Y	Y	*Y*	Y	Y	Y	Y
Intervention(s)	Y	Y	Y	Y	Y	Y	Y	Y	Y	Y	Y	*Y*	Y	Y	Y	Y
Key inclusion and exclusion criteria	Y	Y	Y	Y	Y	Y	Y	Y	Y	Y	Y	*Y*	Y	Y	Y	Y
Study type	Y	Y	Y	Y	Y	Y	Y	N	Y	Y	Y	*Y*	Y	Y	Y	Y
Date of first enrollment	Y	Y	Y	Y	Y	Y	Y	Y	Y	Y	Y	*Y*	Y	Y	Y	Y
Target sample size	Y	Y	Y	Y	Y	Y	Y	Y	Y	Y	Y	*Y*	Y	Y	Y	Y
Recruitment status	Y	Y	Y	Y	Y	Y	Y	Y	Y	Y	Y	*Y*	Y	Y	Y	Y
Primary outcome(s)	Y	Y	Y	Y	Y	Y	Y	Y	Y	Y	Y	*Y*	Y	Y	Y	Y
Key secondary outcomes	Y	Y	Y	Y	Y	Y	Y	Y	Y	Y	Y	*Y*	Y	Y	Y	Y

**Table 3:** The other trial registration items in platforms in WHO ICTRP.

The other trial registration items	ANZCTR	ChiCTR	CRIS	Clinical Trials.gov	CTRI	DRKS	IRCT	ISRCTN.org	JPRN	PAC-TR	NTR	ReBec	TCTR
Last updated date	N	Y	N	Y	Y	N	N	Y	Y	N	Y	Y	Y
Estimated primary completion date	Y	N	Y	Y	N	N	N	Y	Y	Y	Y	Y	Y
Estimated completion date	Y	Y	Y	Y	Y	N	Y	Y	Y	Y	Y	Y	N
Actual date last participant enrolled	Y	N	Y	N	N	Y	N	Y	Y	Y	N	N	N
Current outcome measures	N	Y	N	Y	N	N	Y	Y	N	Y	N	N	N
Outcome time point	Y	Y	Y	N	N	N	Y	N	N	Y	Y	N	N
Reason abandoned	N	N	N	N	N	N	N	Y	N	N	N	N	N
Public acronym	N	N	N	N	N	N	N	N	N	N	N	Y	N
Trial acronym	Y	N	Y	N	N	Y	Y	Y	N	Y	N	Y	N
Brief summary	Y	N	Y	Y	Y	Y	Y	Y	N	N	Y	N	N
Detailed description	Y	N	Y	Y	Y	N	N	N	N	Y	Y	Y	Y
Additional inclusion criteria	N	N	N	N	N	Y	N	Y	N	N	N	N	N
Actual sample size	Y	Y	N	Y	N	N	N	N	N	Y	N	N	Y
Gender	Y	Y	Y	Y	Y	Y	Y	Y	Y	Y	N	Y	Y
Age	N	N	Y	Y	N	N	N	Y	N	N	Y	N	Y
Minimum age	Y	Y	N	N	Y	Y	Y	N	Y	Y	N	Y	Y
Maximum age	Y	Y	N	N	Y	Y	Y	N	Y	Y	N	Y	Y
Accept healthy volunteers	Y	N	Y	Y	N	N	N	N	N	N	N	N	Y
Condition category	Y	N	N	N	Y	N	Y	Y	N	N	N	N	N
Condition code	Y	Y	N	N	N	Y	Y	N	N	N	N	N	N
Removed location countries	N	N	N	Y	N	Y	N	Y	N	N	N	N	N
Study design	N	Y	N	N	Y	N	N	Y	Y	N	N	N	N
Allocation	Y	N	Y	N	N	Y	N	N	Y	Y	Y	Y	Y
Blinding	Y	Y	Y	Y	Y	Y	Y	N	Y	Y	Y	Y	Y
Who is blinded	Y	Y	Y	N	N	Y	N	N	N	N	N	N	N
Allocation concealment procedures	Y	N	N	N	Y	N	N	N	Y	Y	N	N	N
Sequence generation	Y	Y	N	N	Y	N	N	N	N	Y	N	N	N
Randomization	N	N	N	Y	N	N	Y	N	Y	N	Y	N	N
Intervention model	Y	Y	Y	Y	N	N	Y	N	N	N	Y	Y	Y
Intervention type	N	N	Y	N	N	N	N	Y	Y	Y	N	N	N
Purpose	Y	Y	Y	Y	N	Y	Y	N	Y	Y	N	Y	Y
Assignment	Y	N	Y	Y	N	Y	Y	N	N	Y	N	Y	Y
Phase	Y	Y	Y	Y	Y	Y	Y	Y	Y	N	N	Y	Y
Drug names	N	N	N	N	N	N	Y	Y	N	N	N	N	N
Comparator/control treatment	Y	N	N	N	N	Y	N	N	N	Y	Y	N	N
Other design features	Y	N	N	N	N	N	Y	N	N	N	N	N	N
Study Arm (s)	Y	Y	Y	Y	N	Y	N	N	Y	Y	Y	N	Y
Number of Arms	N	N	Y	N	N	N	N	N	Y	N	N	Y	Y
Publications	Y	N	Y	Y	Y	N	N	Y	Y	N	Y	N	N
Information provided by	N	Y	Y	Y	N	N	Y	N	N	N	N	Y	N
Other related information	Y	N	N	N	N	N	N	Y	Y	N	N	N	N
Ethics application status	Y	N	Y	N	N	N	N	Y	N	Y	N	N	N
Ethic committee information	Y	Y	Y	N	Y	Y	Y	N	N	Y	N	Y	N
Data monitoring committee	N	N	Y	N	N	N	N	N	N	N	N	Y	N
Change history	N	N	N	Y	N	N	N	N	N	N	Y	N	Y

**Table 4:** The number of clinical trials of acupuncture-moxibustion in WHO ICTRP.

Category	Characteristic	Number of *n* = 425 (%)
Register time (year)	2013	124
	2014	123
	2015	178

Study type	Intervention study	379
	Observation study	14
	Others	32

Study design	With control group	374
	Sham acupuncture in control group	164
	Acupuncture versus nonacupuncture (e.g., pharmacologic treatment, physiotherapy)	163
	The other treatment program	97

Countries of recruitment	China	187^*∗*^
	United States	50
	Korea	47
	Other Countries	120
	Unclear	21

Sample size	Maximum/minimum	1000/4

^*∗*^Including Hongkong and Taiwan.

**Table 5:** The number of clinical trials of acupuncture-moxibustion in 16 countries in WHO ICTRP.

	Countries/district	Internet site	The number of clinical trials in acupuncture	The number of clinical trials In moxibustion	The others	Total number
*Platform*						
Clinical Trials.gov	United States	http://www.clinicaltrials.gov/	178 (176 + 2^*∗*^)	3 (1 + 2^*∗*^)	22	201
ChiCTR	China	http://www.chictr.org.cn/	99	3	4	106
CRIS	Kore	http://cris.nih.go.kr/	28 (27 + 1^*∗*^)	2 (1 + 1^*∗*^)	1	30
IRCT	Iran	http://www.irct.ir/	13	0	8	21
ANZCTR	Australia/New Zealand	http://www.anzctr.org.au/	8	0	4	12
ISRCTN.org	United Kingdom	http://www.isrctn.org/	16	0	1	17
JPRN	Japan	http://rctportal.niph.go.jp/	16 (14 + 2^*∗*^)	4 (1 + 2^*∗*^)	0	18
DRKS	Germany	http://www.germanctr.de/	4	0	1	5
ReBec	Brazil	http://www.ensaiosclinicos.gov.br/	5	0	1	6
TCTR	Thailand	http://www.clinicaltrials.in.th/	6	0	0	6
NTR	Netherlands	http://www.trialregister.nl/	1	0	0	1
PAC-TR	Africa	http://www.pactr.org/	1	0	0	1
CTRI	India	http://www.ctri.nic.in/	1	0	0	1

*Total*						*425*

^*∗*^Report both acupuncture and moxibustion in titles.

**Table 6:** The condition focus on in the trails in ICTRP.

Condition focused on in the studies	Acupuncture	Moxibustion	The others	Total number
Pain	75	1	6	82
Cancer	28	0	3	31
Stroke	17 (16 + 1^*∗*^)	1^*∗*^	0	17
Arthritis	11	0	4	15
Depression	11	0	2	13
Insomnia	11	0	0	11
Polycystic ovarian syndrome	9	0	1	10
Obesity	7	0	2	9
Hypertension	6	0	1	7
The others	208 (202 + 6^*∗*^)	11 (5 + 6^*∗*^)	24	237
*Total*	*376*	*6*	*43*	425^#^

^*∗*^Report both acupuncture and moxibustion in titles. ^#^Seven trials with 2 diseases in titles (2 with pain + cancer, 2 with cancer + insomnia, 2 with stroke + depression, 2 with depression + insomnia).

**Table 7:** The characteristic information of clinical trials of acupuncture-moxibustion in WHO ICTRP.

	Number of needle insertions per subject per session (mean and range where relevant) *n* (%)	Names (or location if no standard name) of points used (uni/bilateral) *n* (%)	Depth of insertion, based on a specified unit of measurement, or on a particular tissue level *n* (%)	Response sought (e.g., de qi or muscle twitch response) *n* (%)	Needle stimulation (e.g., manual, electrical) *n* (%)	Needle retention time *n* (%)	Needle type (diameter, length, and manufacturer or material) *n* (%)	Number of treatment sessions *n* (%)	Frequency and duration of treatment sessions *n* (%)	Total number
*Platform*										
ANZCTR	0	5 (41.7)	0	2 (16.7)	7 (58.3)	7 (58.3)	9 (75.0)	4 (33.3)	7 (58.3)	12
ChiCTR	2 (1.9)	18 (17.0)	2 (1.9)	4 (3.8)	35 (33.01)	12 (11.3)	5 (4.7)	6 (5.7)	9 (8.5)	106
CRIS	5 (16.7)	23 (76.7)	6 (20.0)	5 (16.7)	8 (26.7)	16 (53.3)	17 (56.7)	13 (43.3)	25 (83.3)	30
Clinical Trials.gov	12 (6.0)	93 (46.3)	37 (18.4)	23 (11.4)	75 (37.3)	85 (42.3)	82 (40.8)	56 (27.9)	90 (44.8)	201
CTRI	0	0	0	0	0	1	0	0	1 (100.0)	1
RPCEC	0	0	0	0	0	0	0	0	0	0
DRKS	0	0	0	0	1 (20.0)	1 (20.0)	2 (40.0)	2 (40.0)	2 (40.0)	5
IRCT	1 (4.8)	11 (52.3)	1 (4.8)	0	1 (4.8)	11 (52.3)	3 (14.3)	2 (9.5)	8 (38.1)	21
ISRCTN.org	0	9 (52.9)	4 (23.5)	3 (17.6)	2 (11/8)	5 (29.4)	4 (23.5)	5 (29.4)	11 (64.7)	17
JPRN	0	3 (16.7)	0	0	6 (33.3)	1 (5.6)	0	2 (11.1)	2 (11.1)	18
PAC-TR	0	0	0	0	0	0	0	1 (100.0)	1 (100.0)	1
SLCTR	0	0	0	0	0	0	0	0	0	0
NTR	0	0	0	0	0	0	0	0	0	0
ReBec	1 (16.7)	4	0	0	2 (33.3)	4 (66.7)	1 (16.7)	4 (66.7)	4 (66.7)	6
EU-CTR	0	0	0	0	0	0	0	0	0	0
TCTR	0	3 (50.0)	0	1 (16.7)	1 (16.7)	2 (33.3)	3 (50.0)	0	2 (33.3)	6
*Countries of recruitment*										
China	3 (1.6)	65 (34.8)	20 (10.7)	15 (8.0)	74 (39.6)	55 (29.4)	32 (17.1)	30 (16.0)	60 (32.1)	187^*∗*^
United States	7 (14.0)	14 (28.0)	2 (4.0)	1 (2.0)	11 (22.0)	13 (26.0)	23 (46.0)	17 (34.0)	21 (42.0)	50
Korea	7 (14.9)	36 (76.6)	12 (25.5)	8 (17.0)	17 (36.2)	25 (53.2)	25 (53.2)	18 (38.3)	35 (76.6)	47
Other countries	3 (2.5)	43 (35.8)	12 (10.0)	10 (8.3)	30 (25.0)	41 (34.2)	38 (31.7)	26 (21.7)	37 (30.8)	120
Unclear	1 (4.8)	11 (52.4)	4 (19.0)	4 (19.0)	6 (28.6)	11 (52.4)	8 (38.1)	4 (19.0)	9 (42.9)	21
*Total*	*21 (4.9)*	*169 (39.8)*	*50 (11.8)*	*38 (8.9)*	*138 (32.5)*	*145 (34.1)*	*126 (29.6)*	*95 (22.4)*	*162 (38.1)*	*425*

^*∗*^Including Hongkong and Taiwan.

**Table 8:** The information in acupuncture-moxibustion in detail about acupuncture manipulation/needle type.

Platform	Acupoint	acupressure	Acupuncture	Auricular acupressure	Auricular acupuncture	Battlefield acupuncture	Dry cupping	Dry needling	Electrical stimulation	Electroacupuncture	Heat therapy	Laser acupuncture	moxibustion	Pharmaco-acupuncture
ANZCTR	0	0	3	0	0	0	0	2	0	3	1	3	0	0
ChiCTR	5	2	64	0	0	0	0	0	0	30	0	2	3	0
CRIS	0	0	22	0	0	0	0	0	0	5	0	1	2	0
Clinical Trials.gov	0	7	102	2	9	3	0	16	0	43	2	11	2	1
CTRI	0	1	0	0	0	0	0	0	0	0	0	0	0	0
RPCEC	0	0	0	0	0	0	0	0	0	0	0	0	0	0
DRKS	0	0	2	0	2	0	0	0	0	0	0	1	0	0
IRCT	0	6	10	0	2	0	2	0	0	1	0	0	0	0
ISRCTN.org	0	1	13	0	1	0	0	0	0	2	0	0	0	0
JPRN	0	0	9	0	0	0	0	0	0	6	0	0	3	0
PAC-TR	0	0	0	0	0	0	0	0	0	0	0	0	1	0
SLCTR	0	0	0	0	0	0	0	0	0	0	0	0	0	0
NTR	0	0	1	0	0	0	0	0	0	0	0	0	0	0
ReBec	0	1	1	0	1	0	0	0	0	3	0	0	0	0
EU-CTR	0	0	0	0	0	0	0	0	0	0	0	0	0	0
TCTR	0	0	3	0	0	0	0	2	0	1	0	0	0	0
*Total*	*5*	*18*	*229*	*2*	*15*	*3*	*2*	*20*	*0*	*96*	*3*	*18*	*13*	*1*

**Table 9:** The information about sham acupuncture in control group in register clinical trials of acupuncture-moxibustion.

	The study design in control group			Operational approach in details of sham acupuncture					
	Acupuncture versus the other interventions without acupuncture (e.g., drugs and physiotherapy)	Acupuncture versus the other interventions (e.g., different acupuncture method)	Acupuncture versus sham acupuncture	Names of points	Material	Stimulation approach	Depth of insertion	Duration of treatment sessions	No related information
*Platform*									
ANZCTR	3	3	6	0	0	0	0	0	6
ChiCTR	41	16	49	20	0	8	0	0	21
CRIS	12	9	9	2	0	0	1	0	6
Clinical Trials.gov	81	46	74	23	1	13	15	0	22
CTRI	1	0	0	0	0	0	0	0	0
RPCEC	0	0	0	0	0	0	0	0	0
DRKS	1	3	1	0	0	0	0	0	1
IRCT	11	2	8	7	0	0	0	1	0
ISRCTN.org	4	6	7	5	0	0	1	0	1
JPRN	3	8	7	2	0	2	0	0	3
PAC-TR	1	0	0	0	0	0	0	0	0
SLCTR	0	0	0	0	0	0	0	0	0
NTR	0	0	1	0	0	0	1	0	0
ReBec	3	1	2	0	0	0	1	0	1
EU-CTR	0	0	0	0	0	0	0	0	0
TCTR	2	4	0	0	0	0	0	0	0
*Countries of recruitment*									
China	63	31	78	32	1	12	2	0	31
United States	20	18	12	5	0	1	3	0	3
Korea	22	9	16	5	0	2	1	0	8
Other countries	49	38	49	14	0	8	8	1	18
Unclear	9	2	9	3	0	0	5	0	1
*Total*	*163*	*98*	*164*	*59*	*1*	*23*	*19*	*1*	*61*
